# Low-Refractive-Index SiO_2_ Nanocolumnar Thin Films Fabricated by Oblique Angle Deposition

**DOI:** 10.3390/ma18102225

**Published:** 2025-05-12

**Authors:** Bojie Jia, Gang Chen, Sheng Zhou, Xiaofeng Ma, Qiuyu Zhang, Yujia Geng, Teng Xu, Dingquan Liu

**Affiliations:** 1 Shanghai Key Laboratory of Optical Coatings and Spectral Modulation, Shanghai Institute of Technical Physics, Chinese Academy of Sciences, Shanghai 200083, China; jiabojie22@mails.ucas.ac.cn (B.J.); gangchen@mail.sitp.ac.cn (G.C.); xuteng2023@shanghaitech.edu.cn (T.X.); 2 University of Chinese Academy of Sciences, Beijing 100049, China; 3 School of Physical Science and Technology, ShanghaiTech University, Shanghai 200031, China

**Keywords:** low refractive index, SiO_2_ films, oblique angle deposition, nanocolumnar structure

## Abstract

The refractive index is one of the most important optical parameters of optical thin films. Optical films with a low refractive index can effectively reduce the residual reflection on the film surface, which is one of the most important parameters pursued by scholars. In this research, SiO_2_ thin films with a low refractive index and nanocolumnar structures were prepared by oblique angle deposition (OAD). The SiO_2_ thin films deposited at different inclination angles were prepared using the electron beam evaporative deposition method. The single-layer film samples were measured by ellipsometry, infrared spectrometry, scanning electron microscopy (SEM), and atomic force microscopy (AFM). The experimental results demonstrated that at an inclination angle of 85°, the average refractive index of the film decreased to 1.30 in the 350–1300 nm wavelength range. Additionally, the film deposited on one side of a crystalline Al_2_O_3_ substrate achieved a transmittance of 92.1% in the 350–1500 nm wavelength range and the residual reflectance was reduced by 0.7%.

## 1. Introduction

In order to overcome the influence of Fresnel reflection on optical surfaces and improve the transmittance and optical performance of optical elements in the working band, anti-reflection (AR) coatings have been developed for a long time. AR coatings (thin films) have been applied extensively in optical elements, optoelectronic devices, instruments, and systems [1,2]. Anti-reflection thin films are one kind of the most common optical thin films, which are used to improve the energy transfer efficiency of optical systems and to reduce stray light. Currently, widely used broadband AR films include multilayer films composed of high- and low-refractive-index layers, as well as single-layer AR coatings with an appropriate refractive index and optical thickness [3,4]. As the broadband and wide-angle characteristics of interference multilayers can be effectively improved by using an outermost layer with a lower refractive index, reducing the effective refractive index of the outermost layer has always been a research priority.

Because of its optical properties of high transparency and low refractive index, nanostructured silicon dioxide has been extensively studied for AR coatings [5,6]. Nanostructures can provide larger porosity, reducing the refractive index of films and the amount of reflected light in a wide wavelength range and all incident angles [7]. Methods for preparing nanostructured SiO_2_, such as the sol-gel method, produce nanoporous SiO_2_ thin films with a low refractive index, good mechanical strength, and low scattering coefficient [8,9,10,11,12]. However, it is difficult to accurately control the film thickness and uniformity during the spin-coating process, which makes it unsuitable for fine optics and commercial applications. A physical vapor deposition (PVD) method using an oblique angle deposition (OAD) technique is an alternative method of choice. OAD is a technique that enables the growth of porous and sculptured films by utilizing the self-shadowing effect during the deposition process. Nanostructured thin films with controllable porosity and shape can be prepared by utilizing the self-shadowing effect and surface diffusion during the deposition process [13,14,15,16,17,18,19,20].

Using the OAD technique, F. Cao et al. [21,22] deposited SiO_2_ nanocolumnar films on fused silica glass, which achieved a refractive index below 1.20 at a deposition angle of 80°. Researchers such as F. Maudet [23] and J. C. Zhang [24] fabricated multilayer SiO_2_ nanocolumnar thin films on BK7 glass using OAD, achieving excellent AR performance and high transmittance, along with good broadband and wide-angle AR properties. However, these films exhibited relatively complex structures, high porosity, and limited overall stability.

All of the above studies obtained SiO_2_ nanocolumnar structured films with a low refractive index, but there are few studies on the wavelength dependence of the refractive index of films in the visible and near-infrared wavelength bands. In this paper, SiO_2_ nanocolumnar structured films were prepared using the electron beam evaporative OAD method, and the basic properties of the films, such as the refractive index variation, spectral properties, surface morphology, cross-sectional structure, and robustness, were tested and analyzed.

## 2. Experimental Details

### 2.1. Sample Preparation

**Figure 1:**
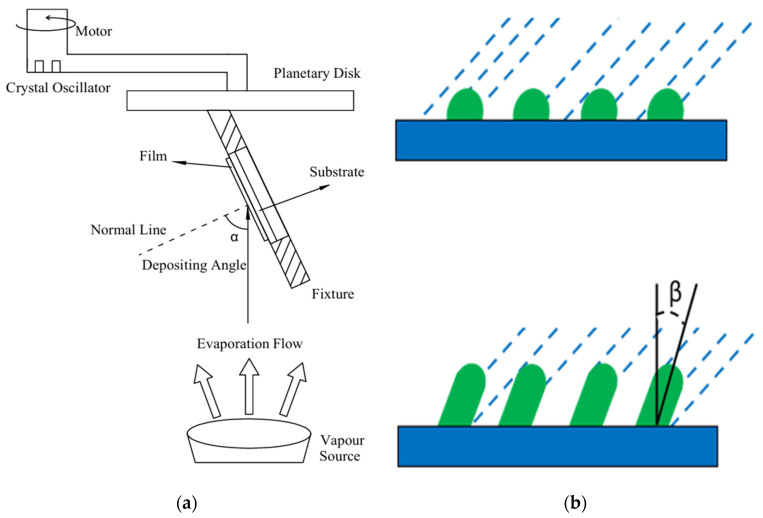
(**a**) Schematic diagram of the principle of the OAD experiment; (**b**) Schematic diagram of the shadowing effect at the beginning of deposition and subsequent growth.

All samples were prepared by electron beam evaporative deposition. According to the actual conditions, the experiments were carried out by designing fixtures with different inclination angles to realize oblique angle deposition coating. SiO_2_ films were prepared on single-side polished Si substrates and transparent Al_2_O_3_ substrates at different inclination angles (0°, 25°, 45°, 55°, 65°, 75°, and 85°) using beveled fixtures. The diameter of the substrates was 25 mm. The Si and Al_2_O_3_ substrates were cleaned with a mixed solution of ethanol and ether (volume ratio 3:1), dried, placed in beveled fixtures, and then loaded into the vacuum deposition chamber. The inclination angle between the normal of the substrate and the direction of vapor incidence, i.e., the OAD inclined angle, is shown in Figure 1a. The source SiO_2_ material used was of 99.99% purity and 0.5~1 mm particle size (produced by Kunshan Guangming Optoelectronic Components Company, Kunshan, China). The distance between the vapor source and the substrate was about 80 cm. The vacuum pressure was pumped below $1.5\times 10^{-2}\;Pa$, and the temperature of the vapor source was about 2000~2200 °C. The thin films were deposited at a natural temperature of about 50~60 °C without heating, and the deposition rate was controlled at about 1 nm/s.

Figure 1b illustrates the most basic concept of obliquely deposited thin films: the shadowing effect [13]. It can be seen that the first set of crystal particles formed in the earliest stage of deposition cast part of a shadow behind them, which prevented any subsequent evaporation from being deposited in these “shadow” regions. Obviously, there is a certain relationship between the inclination angle (β) of the nanocolumn shown in Figure 1b and the inclination angle (α) of the OAD shown in Figure 1a. The tangent formula [25] and the cosine formula [26] are the two most popular heuristic expressions at present, which relate the inclination angle of evaporation with that of the nanocolumn, as follows:

(1) tan⁡α=2tan⁡β

(2) β=α−arcsin⁡(1−cos⁡α)/2

Understanding the fundamental factors controlling the inclination angle of the nanocolumn remains an unsolved question, although other more sophisticated empirical descriptions have been proposed. Zhou et al. [27] made a systematic study on various materials, which clearly showed that the inclination angle of nanocolumns is a material-dependent property, so it cannot be attributed to simple geometrical relationships alone. Therefore, in this paper, the inclination angle of the nanocolumn is not studied in depth.

### 2.2. Measurement

**Figure 2:**
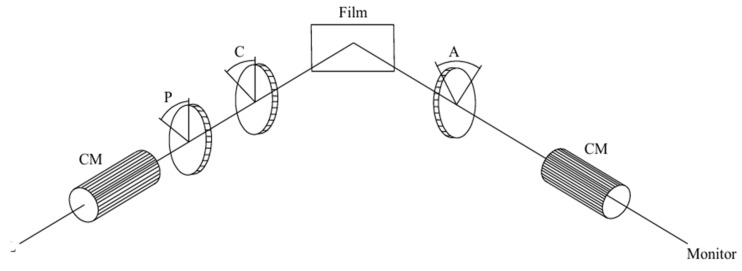
Measuring schematic diagram of ellipsometer.

At the three incident angles of 65°, 70°, and 75°, the ellipsometric parameters ∆ and ψ were measured using an ellipsometer (V-VASE, J.A. Woollam, Lincoln, NE, USA) for the samples in the spectral wavelength range of 350–1300 nm with a step size of 5 nm. A schematic diagram of the ellipsometer is shown in Figure 2. L is the light source, CM is the collimating lens set, P is the polarizer, C is the compensator, Film is the measurement sample, A is the analyzing film, and finally the detector is connected. When the incident light passes through the film sample, its reflectivity and phase will be changed. Assuming that the reflectivities are $r_{p}$ and $r_{S}$, respectively, a set of ellipsometric coefficients (∆ and ψ) can be obtained from the detector by adjusting P, C, and A. ψ represents the real value of the ratio of the complex reflectance coefficients for the s-components and p-components of the outgoing light, and ∆ represents the phase difference between the electric field reflection components $E_{rp}$ and $E_{rs}$. The ellipsometric parameters directly reflect the change in the polarization state of light before and after reflection. The formulae for ∆ and ψ are given by Equations (3)–(5):

(3) $$\rho =\frac{r_{p}}{r_{S}}=\frac{\left|r_{p}\right|e^{i\delta_{p}}}{\left|r_{S}\right|e^{i\delta_{s}}}=\tan\psi e^{i∆}$$

(4) $$∆=\delta_{p}-\delta_{s}$$

(5) $$\psi =\tan^{-1}\left|\frac{r_{p}}{r_{s}}\right|$$

Therefore, the measured ellipsometric parameters ∆ and ψ can be used to invert the refractive index, extinction coefficient, and thickness of the film using multi-beam interference theory and the Fresnel formula. In the ellipsometer’s dedicated V-VASE32 software (version number: 3.736), the optical constant and film thickness data of SiO_2_ films are obtained by inputting data, establishing a film model, and selecting an appropriate dielectric model for fitting to obtain smaller mean squared error (MSE) values.

For the accuracy of ellipsometer measurement, the sample substrates tested were all single-side polished silicon wafers. Due to different inclination angles during the preparation process of the same batch of samples, the deposition efficiency of the films on the substrate varied. As a result, the thickness of the films at different inclination angles within the same batch were also different, with larger inclination angles resulting in thinner films. Table 1 summarizes the data for the samples obtained through ellipsometer measurements.

The transmission spectra of the SiO_2_ films on the Al_2_O_3_ substrate were measured using a spectrophotometer (Lambda 900, PerkinElmer, Waltham, MA, USA) over a wavelength range of 350–1500 nm, with an accuracy of 0.08 nm in the UV–Visible region and 0.3 nm in the near-infrared (NIR) region guaranteed. The parameters of the samples tested by spectrophotometry were consistent with those in Table 1, except that the substrate was Al_2_O_3_. Due to the highly porous nature of the films grown by inclined deposition, water absorption would theoretically increase the refractive index, which would adversely affect the measurements. Additionally, the absorption caused by the water absorbed by the porous films would also lead to additional optical losses and reduce the transmittance of the films. Therefore, the effect of water absorption should be eliminated as much as possible during the measurement. All samples were baked in an air oven at 110 °C to remove any moisture before the measurement in order to obtain data that closely reflected the properties of the pure film layer.

The cross-sectional morphology of the SiO_2_ nanocolumnar films and their actual thickness were examined using a Verios field emission scanning electron microscope (Thermo Fisher, Waltham, MA, USA). The surface morphology and surface roughness of the films were observed using an FM-Nanoview6800 ( Flyingman Precision Instruments, Suzhou, China) atomic force microscope (AFM).

## 3. Results and Discussion

### 3.1. Optical Performance

**Figure 3:**
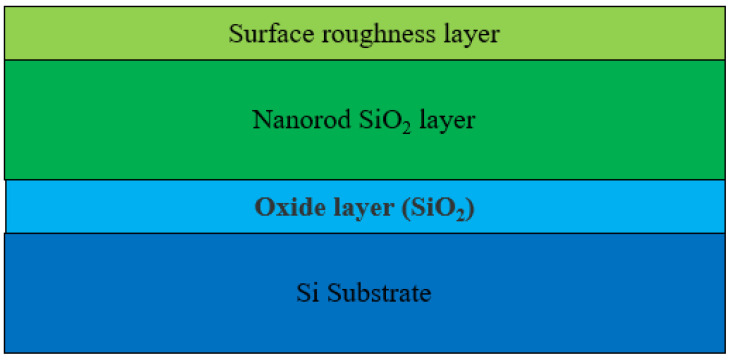
Ellipsometric structural model of SiO_2_ film.

**Figure 4:**
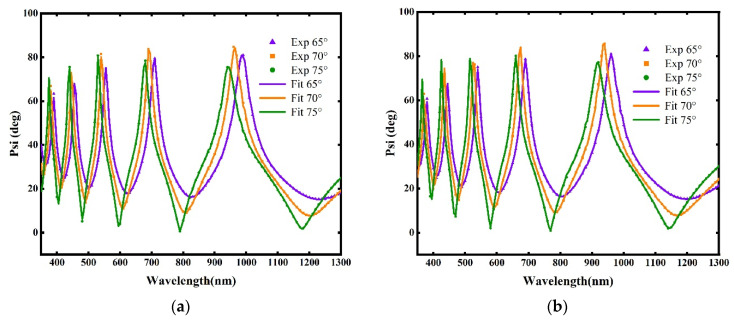

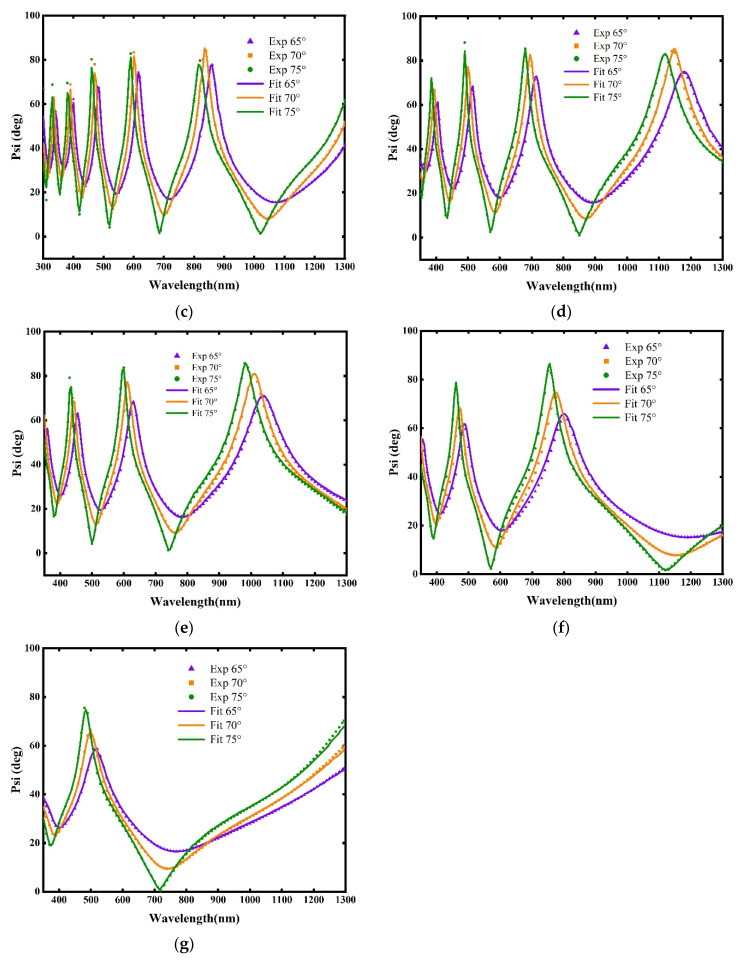
The fitted and measured ellipsometric parameters (Psi) for: (**a**) inclination angle 0°, (**b**) inclination angle 25°, (**c**) inclination angle 45°, (**d**) inclination angle 55°, (**e**) inclination angle 65°, (**f**) inclination angle 75°, and (**g**) inclination angle 85°.

Based on the ellipsometric structural model shown in Figure 3, the ellipsometric parameters of the seven samples summarized in Table 1 were measured and fitted by ellipsometry. The Sellmeier model and Tauc–Lorentz model were used to describe the refractive index of the SiO_2_ thin films. The Sellmeier model demonstrated good accuracy in the VIS–NIR spectrum while the Tauc–Lorentz model showed better performance in the near UV region (350–400 nm). Figure 4 shows the ellipsometric fitting results for the seven samples. It can be seen that the data obtained from the structural model fitting were in good agreement with the experimental data measured using the ellipsometer. This indicated that the refractive index of the SiO_2_ thin films obtained from the fitting had good accuracy.

**Figure 5:**
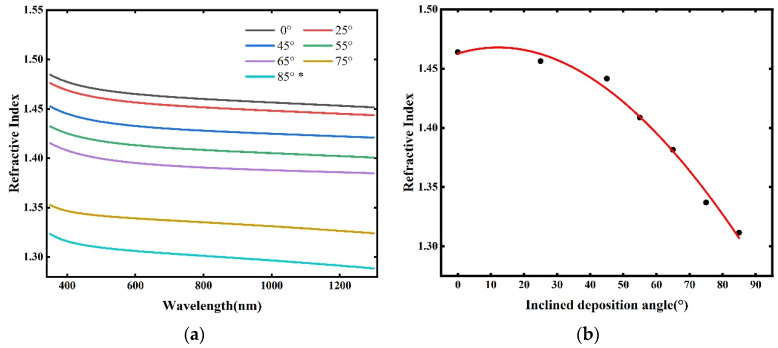
Under different deposition angles: (**a**) schematic of the refractive index variation with wavelength for the samples; and (**b**) refractive index fitting curve of the nanocolumnar thin films at the 640 nm wavelength. * Inclined deposition angles.

The optical parameters of the thin films, including the refractive index and extinction coefficient, can be obtained from the results of ellipsometric parameter fitting. However, the presence of voids leads to the determination of an effective refractive index rather than the intrinsic material index. The extinction coefficient data at the 640 nm wavelength are summarized in Table 2. It shows that the calculated values of the extinction coefficients of the SiO_2_ thin films were lower than 5 × 10^−5^, which means that the SiO_2_ thin films were characterized by very low optical losses and could be regarded as highly transparent thin films. Figure 5a presents the wavelength dependence of the refractive index of the SiO_2_ films at different inclination angles. From that figure, it can be observed that the refractive index curves aligned well with the actual refractive index curves of the SiO_2_ films, which validated the accuracy of the fitted data. The refractive index curves of the SiO_2_ films kept moving downward with the gradual increase in the inclination angle using the oblique angle deposition method, which means that the increase in the OAD inclination angle led to the decrease in the refractive index of the SiO_2_ films. The refractive index of the SiO_2_ films deposited at different inclination angles all showed a decrease. What is worth mentioning is that the refractive index of the SiO_2_ films successfully dropped to around 1.31 in the visible light range and approximately 1.30 in the near-infrared wavelength band. Using the data of the refractive index curve in Figure 5a and fitting with cubic curve, the fitting curve of the refractive index of the SiO_2_ films at 640 nm with the inclination angle is shown in Figure 5b. The variation in the refractive index within the inclination angle range of 0° to 85° can be approximately calculated using Equation (6):

(6) $$n={5.13\times 10}^{-8}\theta^{3}-3.60\times 10^{-5}\theta^{2}+{8.54\times 10}^{-4}\theta +1.46$$

In the inclination angle range of 0° to 85°, the refractive index of the SiO_2_ films exhibited a cubic relationship with the inclination angle. As the inclination angle increased, the decrease in the refractive index with respect to the angle became more pronounced. In conclusion, increasing the inclination angle during oblique angle deposition effectively reduced the refractive index of the SiO_2_ films.

**Figure 6:**
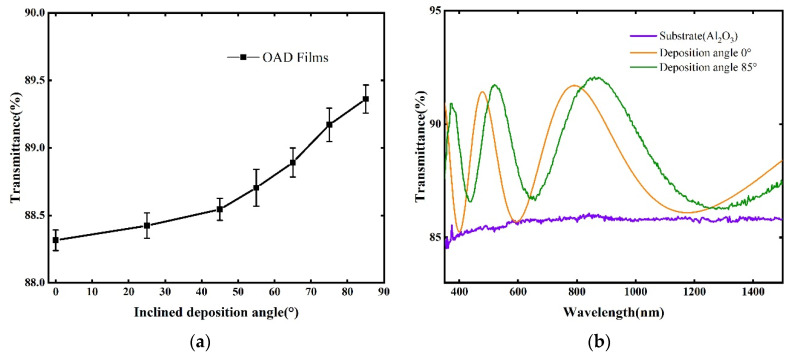
Average transmittance of thin film samples in the 350–1500 nm wavelength range: (**a**) under different OAD inclination angles; and (**b**) transmittance curves of the substrate and thin film samples deposited at inclination angles of 85° and 0°.

The average transmittance curves of the SiO_2_ nanocolumnar thin films deposited at different inclination angles are shown in Figure 6a with respect to the inclination angle in the wavelength range of 350–1500 nm. It can be observed that the SiO_2_ nanocolumnar thin films prepared using the OAD method had a certain transmittance enhancement effect compared to the conventional SiO_2_ films, and the transmittance enhancement effect of the films increased with the increase in the inclination angle accordingly. This phenomenon can be explained by the principle of AR coatings. In AR coatings, the refractive index and thickness are carefully designed to minimize the reflection of light at the film–substrate interface. For the SiO_2_ nanocolumnar films prepared using the OAD method, the porous and nanostructured nature of the films, along with the varying inclination angle, helped to reduce refection by creating a gradient in the refractive index, which enhanced light transmission through the films. Therefore, as the inclination angle increased, the film’s structure became more optimized for light passage, thereby improving its anti-reflection properties and increasing transmittance. In addition, the variation in the film’s transmittance became more pronounced as the inclination angle increased. This trend can be attributed to the increased structural anisotropy and porosity of the films at high inclination angles, which further optimized light transmission by reducing surface reflection and scattering, thereby enhancing the overall transmittance.

The transmittance curve of the SiO_2_ nanocolumnar film prepared on the Al_2_O_3_ substrate with an inclination angle of 85° in the wavelength range of 350–1500 nm is plotted in Figure 6b, and the transmittance curve of the substrate is also plotted for reference. Then, the spectrum of a conventional SiO_2_ thin film with the same thickness as the sample is added for comparison, which shows that the SiO_2_ nanocolumnar film had a higher transmittance than that of the conventional SiO_2_ thin film. The reasons, as shown in Figure 1b, were due to the shadowing effect, the thin films deposited at an inclined angle, and the nanocolumnar structures. During the thin film deposition process, the initially deposited material particles formed shadow areas that prevented subsequent particles from being deposited in these areas. As a result, subsequent material particles could only be deposited on higher areas of the thin film surface. Besides, the atoms deposited at natural temperature did not have sufficient energy to diffuse into the shadow areas; thus, columnar structures were formed. The larger the inclination angle of deposition, the more pronounced the shadow effect. In addition, the density of the thin films gradually decreased from the substrate to the thin film surface due to the shadowing effect induced by the OAD method. Therefore, in fact the refractive index of the SiO_2_ nanocolumnar thin films was not uniform. This non-uniformity of the refractive index led to a shift in the spectral curve of the thin films, causing it not to intersect with the substrate’s spectral curve. Additionally, due to the difference in refractive indices, the SiO_2_ nanocolumnar thin films had a lower refractive index compared to conventional SiO_2_ films of the same thickness.

### 3.2. Surface and Internal Structure

According to the Bruggeman model, a porous semiconductor material can be considered as a mixture of two phases, and its effective refractive index $\left(n_{eff}\right)$ in the non-absorbing region follows the equation [28]

(7) $$\;\left(1-\phi \right)\frac{n_{c}^{2}-n_{eff}^{2}}{n_{c}^{2}+2n_{eff}^{2}}+\phi \frac{n_{\phi }^{2}-n_{eff}^{2}}{n_{\phi }^{2}+2n_{eff}^{2}}=0,$$

where $n_{c}$ and $n_{\phi }$ are the refractive indices of the continuous media and pores, respectively; and ϕ is the porosity. As the effect of water was removed during the measurement process, the $n_{\phi }$ value was taken as 1. The films deposited at a 0° incidence angle were considered as continuous media, with the porosity calculated using the refractive index data from Section 3.1. The results are summarized in Table 3. With the increase in the deposition angle, the film porosity gradually increased, reaching the maximum value of 33.7% at 85°.

**Figure 7:**
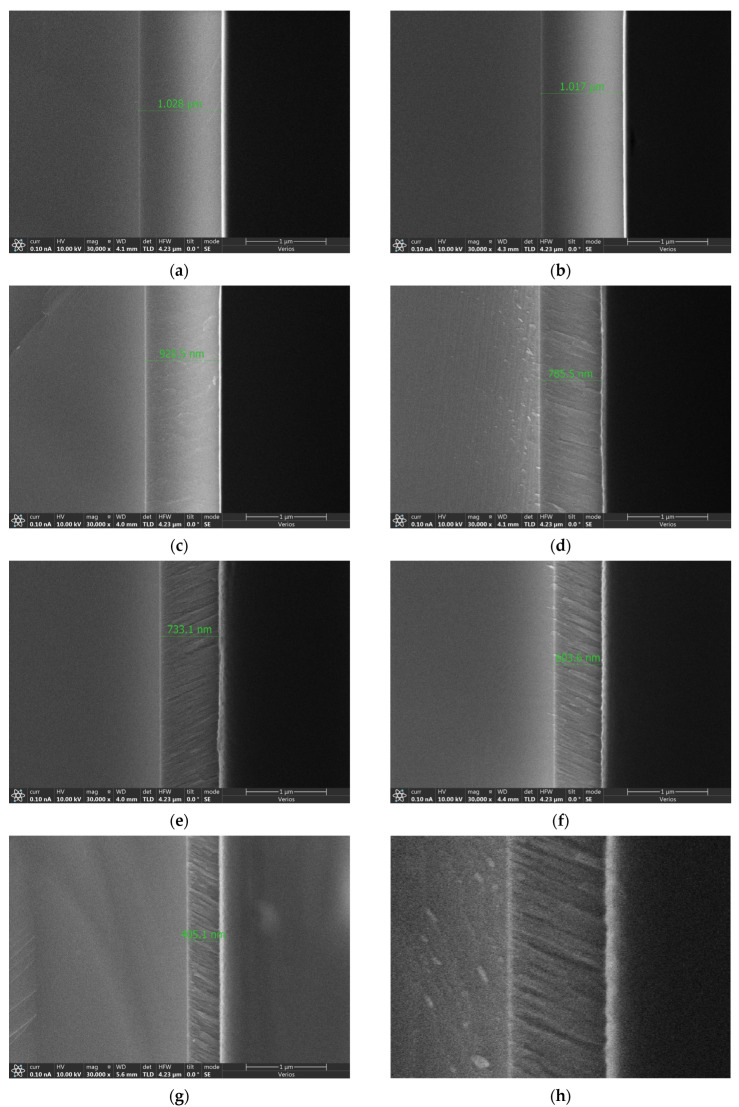
SEM images of samples at different deposition angles: (**a**) inclination angle 0°, (**b**) inclination angle 25°, (**c**) inclination angle 45°, (**d**) inclination angle 55°, (**e**) inclination angle 65°, (**f**) inclination angle 75°, (**g**) inclination angle 85°, and (**h**) amplification of columnar structures.

After sampling the specimens, the cross-sectional morphology of the low-refractive-index SiO_2_ monolayer films were examined using a Verios field emission scanning electron microscope (Thermo Fisher), and the actual film thickness was measured as well. The SEM images obtained from the measurements are shown in Figure 7. It can be clearly seen that when there was no inclination angle (i.e., the inclination angle was 0°), the film appeared dense and possible voids could not be observed. When the inclination angle was 45° or 55°, the SEM images shown in Figure 7c,d revealed the presence of some voids. As the inclination angle increased, the voids became more pronounced, and the number of voids also increased. When the inclination angle increased further, i.e., 65°, 75°, and 85°, the SEM images in Figure 7e–g showed more obvious columnar voids, with denser columnar structures appearing in the films. In addition, in the samples with clearly defined columnar structures, represented by the SEM images of the four samples shown in Figure 7d–g, it can be observed that the angle between the nanocolumn and the substrate gradually increased with the increase in the inclination angle. Figure 7h is an amplification of Figure 7g, revealing clearer nanocolumn morphological details. The larger angle between the nanocolumn and the substrate indicated that the deposition process was more affected by the shadowing effect, resulting in higher porosity in the thin films. This was further confirmed by the SEM images, which showed larger voids between the nanocolumns. These properties were consistent with the description above.

From the SEM images in Figure 7, it was easy to measure the inclination angle (β) of the nanocolumns in the samples. The data of β are summarized in Table 4. The cosine rule described by Equation (2) has certain relevance. And the relationship between α and β can be approximate described by Equation (8):

(8) 2β=α−arcsin⁡(1−cos⁡α)/2

**Figure 8:**
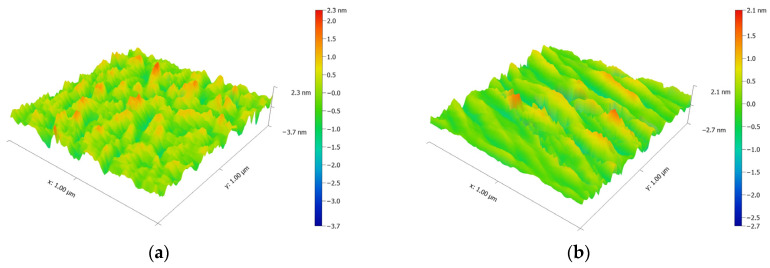

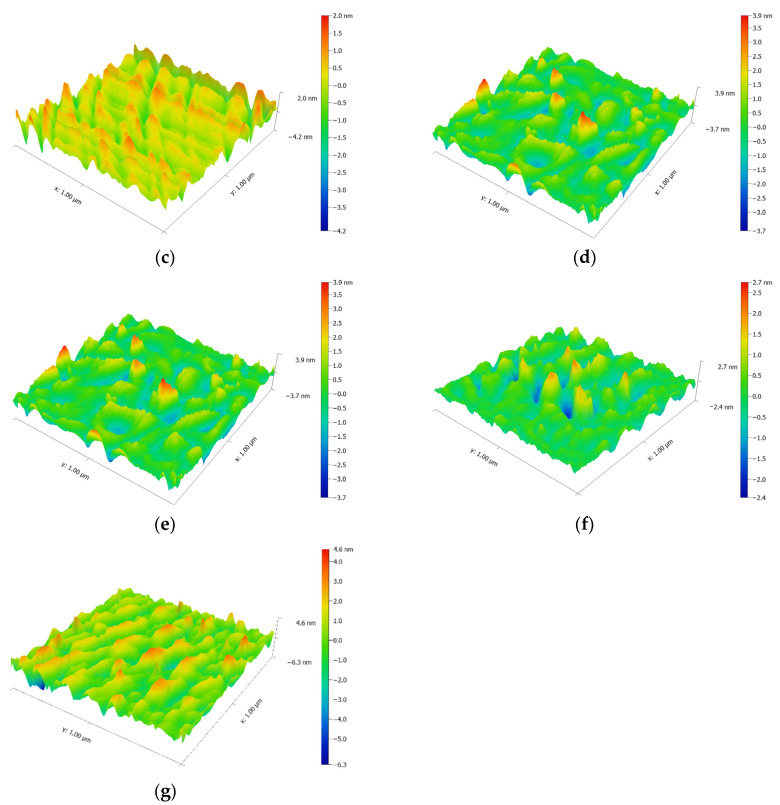
AFM images of SiO_2_ films at different deposition angles: (**a**) inclination angle 0°, (**b**) inclination angle 25°, (**c**) inclination angle 45°, (**d**) inclination angle 55°, (**e**) inclination angle 65°, (**f**) inclination angle 75°, and (**g**) inclination angle 85°.

The surface morphology of the samples was observed by atomic force microscopy (AFM). The obtained surface morphologies are shown in Figure 8. Each image was processed with image editing software (Gwyddion, version number: 2.10) to remove the substrate background effects and adjusted to an angle that best highlighted the surface morphology of the film for easier observation. By comparing the AFM images at different inclination angles in Figure 8, it is clear that when the inclination angle was 0°, the surface of the SiO_2_ film did not exhibit any distinctive morphology. However, when there was a certain tilt angle, the surface of the SiO_2_ films began to display a special morphology, manifested as regularly distributed striped grooves. This pattern aligned with the theoretical top-end morphology of the SiO_2_ nanocolumnar structures, characterized by protrusions at the columnar locations and depressions in the void areas. The AFM images further substantiated the presence of nanocolumnar structures from the perspective of surface properties. Furthermore, due to the porous nature of the OAD films, the scattering will cause additional optical losses, which adversely affect the anti-reflection performance of the films. Therefore, to minimize the scattering as much as possible, the surface roughness and inhomogeneity of the film surface need to be as low as possible as well. By processing the AFM images in Figure 8, the surface roughness of each sample was obtained, and the results are summarized in Table 5. Due to the background influence being removed through fitting during the surface roughness measurements, the calculated surface roughness may be slightly lower. But it was still evident that the surface roughness of the SiO_2_ films increased as the inclination angle increased. In addition, the measured data indicated that the surface roughness of the films deposited by electron beam evaporation was relatively low and, therefore, scattering caused by the surface roughness was relatively insignificant. According to Beckmann’s total integral scattering scalar theory [29], the scattering loss caused by the thin film itself can be considered negligible.

## 4. Analysis and Conclusions

In this research, we prepared low-refractive-index SiO_2_ nanocolumnar films using the OAD method and electron beam evaporative deposition. In the wavelength range of 350–1300 nm, the average refractive index of the SiO_2_ nanocolumnar films deposited at an inclination angle of 85° on the single-side polished silicon substrate was as low as 1.30. This represented a 10% reduction compared to the refractive index of conventional SiO_2_ films, which was 1.445. What’s more, in the wavelength range of 300–1500 nm, the peak transmittance of the SiO_2_ nanocolumnar thin films deposited on the Al_2_O_3_ crystal substrate on one side at the 85° inclination angle reached 92.1%, and the residual reflectivity was reduced by 0.7% on one side. Considering that it was only a one-sided anti-reflection effect, the ideal anti-reflection effect was approached at this wavelength. Therefore, the SiO_2_ nanocolumnar thin films demonstrated a noticeable anti-reflection effect. It was also shown that the refractive index of the thin films decreased and the transmittance increased as the inclination angle increased during deposition. And the cross-section structure and surface morphology of the SiO_2_ nanocolumnar thin films were also investigated. It was found that the samples with larger deposition inclination angles were more affected by the shadowing effect, resulting in thinner films with higher porosity, and more pronounced nanocolumnar structures were observed in these samples. Additionally, it was found that the surface roughness of the SiO_2_ nanocolumnar thin films increased with the inclination angle of deposition.

In summary, the SiO_2_ nanocolumnar thin films fabricated using the OAD method were characterized by a low refractive index and high transmittance. These properties make them promising for applications in AR coatings and other related fields and achieve a very low residual reflection.

## Figures and Tables

**Table 1:** Information of samples.

Substrate	Si	Si	Si	Si	Si	Si	Si
**Deposition angle**	0°	25°	45°	55°	65°	75°	85°
**Film thickness (nm)**	1028	1017	915	785	733	603	408

**Table 2:** Information on the extinction coefficient at the 640 nm wavelength.

Substrate	Si	Si	Si	Si	Si	Si	Si
**Deposition angle**	0°	25°	45°	55°	65°	75°	85°
**Extinction Coefficient**	3 × 10^−7^	5 × 10^−7^	7 × 10^−7^	4 × 10^−6^	6 × 10^−6^	8 × 10^−6^	3 × 10^−5^

**Table 3:** Calculated porosity results of the samples.

Substrate	Si	Si	Si	Si	Si	Si	Si
**Deposition angle**	0°	25°	45°	55°	65°	75°	85°
**Porosity**	0%	4.2%	6.4%	10.6%	19.0%	27.4%	33.7%

**Table 4:** Inclination angle (β) of the nanocolumns in the samples.

Substrate	Si	Si	Si	Si	Si	Si	Si
**Deposition angle**	0°	25°	45°	55°	65°	75°	85°
**Inclination angle β of the nanocolumn**	No obvious nanocolumn			17°	24°	26°	29°

**Table 5:** Surface roughness of samples.

Substrate	Si	Si	Si	Si	Si	Si	Si
**Deposition angle**	0°	25°	45°	55°	65°	75°	85°
**RMS (nm)**	0.447	0.496	0.588	0.790	0.842	0.992	1.128

## Data Availability

The original contributions presented in this study are included in the article. Further inquiries can be directed to the corresponding author.
